# Communication and patient safety in gynecology and obstetrics - study protocol of an intervention study

**DOI:** 10.1186/s12913-019-4579-y

**Published:** 2019-11-28

**Authors:** Sonia Lippke, Julian Wienert, Franziska Maria Keller, Christina Derksen, Annalena Welp, Lukas Kötting, Kerstin Hofreuter-Gätgens, Hardy Müller, Frank Louwen, Marcel Weigand, Kristina Ernst, Katrina Kraft, Frank Reister, Arkadius Polasik, Beate Huener nee Seemann, Lukas Jennewein, Christoph Scholz, Annegret Hannawa

**Affiliations:** 1 Jacobs University Bremen gGmbH, Germany Campus Ring 1, 28759 Bremen, Germany; 2 Die Techniker; Unternehmenszentrale, Fachbereich Versorgungsmanagement, Bramfelder Str. 140, 22305 Hamburg, Germany; 3 Aktionsbündnis Patientensicherheit, Am Zirkus 2, 10117 Berlin, Germany; 4 Klinik für Frauenheilkunde und Geburtshilfe, Universitätsklinikum Frankfurt Goethe-Universität, Theodor-Stern-Kai 7, 60596 Frankfurt am Main, Germany; 5 Universitätsklinikum Ulm, Prittwitzstr. 43, 89075 Ulm, Germany; 6 Universitätsklinikum Ulm, Albert-Einstein-Allee 23, 89070 Ulm, Germany; 7 Center for the Advancement of Healthcare Quality and Patient Safety (CAHQS), Faculty of CommunicationSciences, Università della Svizzera Italiana, Lugano, Switzerland

**Keywords:** Preventable adverse events, Patient safety, Communication competences, Midwifery models of care, HAPA, Participatory intervention development, Digitization, App, eHealth, Health services research

## Abstract

**Background:**

Patient safety is a key target in public health, health services and medicine. Communication between all parties involved in gynecology and obstetrics (clinical staff/professionals, expectant mothers/patients and their partners, close relatives or friends providing social support) should be improved to ensure patient safety, including the avoidance of preventable adverse events (pAEs). Therefore, interventions including an app will be developed in this project through a participatory approach integrating two theoretical models. The interventions will be designed to support participants in their communication with each other and to overcome difficulties in everyday hospital life. The aim is to foster effective communication in order to reduce the frequency of pAEs. If communication is improved, clinical staff should show an increase in work satisfaction and patients should show an increase in patient satisfaction.

**Methods:**

The study will take place in two maternity clinics in Germany. In line with previous studies of complex interventions, it is divided into three interdependent phases. Each phase provides its own methods and data. Phase 1: Needs assessment and a training for staff (*n* = 140) tested in a pre-experimental study with a pre/post-design. Phase 2: Assessment of communication training for patients and their social support providers (*n* = 423) in a randomized controlled study. Phase 3: Assessment of an app supporting the communication between staff, patients, and their social support providers (*n* = 423) in a case-control study. The primary outcome is improvement of communication competencies. A range of other implementation outcomes will also be assessed (i.e. pAEs, patient/treatment satisfaction, work satisfaction, safety culture, training-related outcomes).

**Discussion:**

This is the first large intervention study on communication and patient safety in gynecology and obstetrics integrating two theoretical models that have not been applied to this setting. It is expected that the interventions, including the app, will improve communication practice which is linked to a lower probability of pAEs. The app will offer an effective and inexpensive way to promote effective communication independent of users’ motivation. Insights gained from this study can inform other patient safety interventions and health policy developments.

**Trial registration:**

ClinicalTrials.gov Identifier: NCT03855735; date of registration: February 27, 2019.

## Background

Patient safety is a key target in public health, health services, and medicine [1, 2]. In addition to medical skills and knowledge, *communication* has been shown to be a major contributor to patient safety*,* both within the healthcare team and between healthcare professionals and patients [3–5]. Communication, not only between different professionals, but also between staff members and patients or relatives, is a significant part in clinical routine each day. Important information may be lost because of the use of medical terms and may result in reduced patient safety, especially when medical terms are used with patients and their partners and relatives [3–5].

Different communication errors and barriers to effective communication have been identified in parts of the clinical team. Errors, such as the omission of important information, describe the kind of suboptimal communication behaviors, whereas barriers are obstructions to engaging in effective communication behaviors and include organizational and (inter-) personal factors such as rapidly changing healthcare teams, work overload, lack of mutual respect, not feeling part of the team, lack of self-confidence, and lack of training [6]. In a review [7] it was summarized that “effective clinical communication is respectful, clear, direct, and explicit. Consistent execution of successful communication requires excellent listening skills, superb administrative support, and collective commitment to move past traditional hierarchy and professional stereotyping.”

Studies have shown that in clinical settings, poor communication may be responsible for up to 80% of all preventable adverse events (pAEs; e.g. [4]), which are events affecting patient safety that are caused by unsafe healthcare processes, rather than by the medical condition of the patient [2]. PAEs can occur in all clinical specialties, including *gynecology and obstetrics* [9, 18]. Besides communication between healthcare providers, many studies have demonstrated that *suboptimal doctor-patient communication* is related to pAEs, including medical errors [35]. Not only is it important to communicate or disclose medical errors to patients appropriately [10] but also to communicate (more) safely and effectively in general [11]. In obstetrics, clinical routine is challenging due to high workload and risks of emergency calls (e.g., due to emergency caesarean section) as well as because medical staff is always responsible for both the mother and the fetus/newborn.

A typical example of a pAE in *gynecology and obstetrics* is the confusion of maternal and infant pulse rates during childbirth, which could result in life-long damage to the fetus due to asphyxia during childbirth (damage case analyses by [12]). This pAE may be caused by the ineffective use of available resources due to poor communication between the different partners (staff, patients and their accompanying persons/ social support providers, [13]). PAEs in gynecology and obstetrics include any physical or mental injury to the pregnant woman, fetus, or newborn due to poor communication, which may lead to incorrect medical decisions causing stillbirths, perinatal, neonatal or maternal mortality, unplanned caesarean sections, neonatal morbidity, blood loss, and hemorrhage [8].

With regard to the prevalence of pAEs, an observation study conducted in obstetrics and gynecology by November et al. in Boston, USA, identified forty-one pAEs within a time frame of 12-weeks [14]. In a recent international meta-analysis by Tanaka, Eriksson and Obermair, the incidence of adverse events in gynecological hospital admissions was 10.8% [15]. Of these adverse events, 52.5% could have been prevented and 1.2% resulted in death. The authors note that “preventability can only be assessed from available documentation and can be influenced by study personnel experience, and knowledge [ …] amendments [ …] need to take place to improve healthcare delivery in gynecology” (p. 198f). Improved communication is also likely to result in better healthcare provider and patient outcomes. For instance, a recent study has discussed the importance of communication between healthcare professionals and physician well-being. Physician burnout was associated with an increased risk for unsafe care, unprofessional behaviors, and low patient satisfaction [16–18]. This is especially true when under time constraints [6, 19, 20].

Patient satisfaction is an important patient-centric outcome for a gynecological clinic and its staff. Giving birth can be a positive experience but it can also be painful. The more pain a mother experiences and the longer her convalescence lasts, the more it affects her satisfaction. Despite healthcare providers’ awareness of this relationship, there is still room for improvement in pain treatment during labor [21]. Effective communication is one key in pain management, especially under high stress and demands. Accordingly, this needs to be improved based on empirical evidence. Empirical evidence on the effectiveness of communication training is ambiguous. Several studies show that communication errors and barriers can be reduced by efficient training programs that help individuals to identify, prevent or manage such errors and barriers. For instance, one study demonstrated that a standardized team-training program for healthcare professionals in obstetric units, based on an analysis of common causes for adverse events, was found to successfully raise professionals’ confidence in dealing with complex emergency situations [12]. Perceived ownership of staff has been demonstrated to be the key moderator of the effectiveness of such trainings [22]. However, a recent systematic review concluded that “Current evidence is inadequate to inform content of training or practice“[9]. Thus, communication *between professionals and with patients* should be structured [23, 24] specifically to the specific context. The primary aim of our project, which is theoretically grounded in models of health-related communication [11] and behavioral change (HAPA) [25], is to improve communication behaviors in gynecology and obstetrics. The second aim is to reduce pAEs and increase healthcare provider and patient satisfaction.

### Communication

A multitude of models of communication in healthcare exists. Accurate, clear communication is central to all of them. For instance, communication is one of four central aspects in the TeamStepps framework of teamwork in healthcare, which has been validated in many clinical settings, including obstetrics [26–28]. The communication dimension of this framework utilizes different tools to facilitate communication between healthcare providers, particularly in critical situations like emergencies or handovers. These tools include *check-back* (or closed-loop communication) to ensure the recipient has understood the sender’s information correctly, *callout*, which is used to convey critical information to a larger group of people efficiently, *SBAR* (an acronym standing for *situation*, *background*, *assessment* and *recommendation*), which can be used when requesting help in emergency situations, and a *checklist* for handovers [29]. The importance of sufficient, accurate and clear conveyance of information is also represented in the SACCIA model by Hannawa [11]. The acronym "SACCIA" stands for five core competencies that constitute safe communication in healthcare: "Sufficiency", "Accuracy", "Clarity", "Contextualization" and "Interpersonal Adaptation". The model has been used to classify communication causes of critical incidents, and it explains how communication errors put patient safety at risks [30].

According to the SACCIA model, focusing on transporting factual information is necessary for effective communication, but not sufficient, especially in obstetrics. Communication, and interpersonal/relational dimensions in particular, have also been identified as one of four dimensions in midwifery models of care. In obstetrics, the biomedical or pathogenic approach to patient care adopted by physicians may clash with nurses’ or midwives’ salutogenetic approaches [31]. These models consider that – unlike most other reasons to visit a hospital – birth is a natural process in most cases and a joyful moment for the parents, but it can also cause uncertainty or fear. Thus, consideration of interpersonal or relational aspects of communication with the expectant mother – such as being respectful of and taking her emotions into account – is essential to transport important information to and from the mother and accompanying persons. Moreover, focusing on interpersonal communication with other team members is likely to improve team functioning [32] and thus increase team members’ motivation to engage in more technical communication.

 In addition, the *model of relational coordination (communication between healthcare providers) and relational coproduction (communication between healthcare provider and patient,* [33]*)* is defined as ‘a mutually reinforcing process of communicating and relating for the purpose of task integration’ ([34], p. 301). It combines the technical dimensions of communication (frequent, timely, accurate, and problem-solving) with interpersonal communication goals (shared goals, shared knowledge, and mutual respect [35]). The relational aspect of this model has been adapted to the communicative process of decision-making between midwives and expectant mothers as a response to the critique that informed choice or shared decision models rely too much on factual information without taking the context or conversation partner into account [36].

If the communication competencies are practiced adequately, the quality of healthcare communication is increased and, as a result, risk of pAEs is reduced. [11]. This objective can be achieved through training sessions, and digitization (telehealth, eHealth, mHealth) [37] opens new avenues, especially in times of work concentration due to efficiency increasing, skills shortage due to few experts on the labor market, and multi-tasking as a societal trend [2, 16, 17]. There are multiple advantages to digital training, such as adaptability to the user’s needs [38], just-in-time-interventions [37] and a high number of potential users who are motivated to participate due to technological interests instead of the content, making it easier to reach rather unmotivated individuals [39, 40].

### Behavioral change

**Fig. 1:**
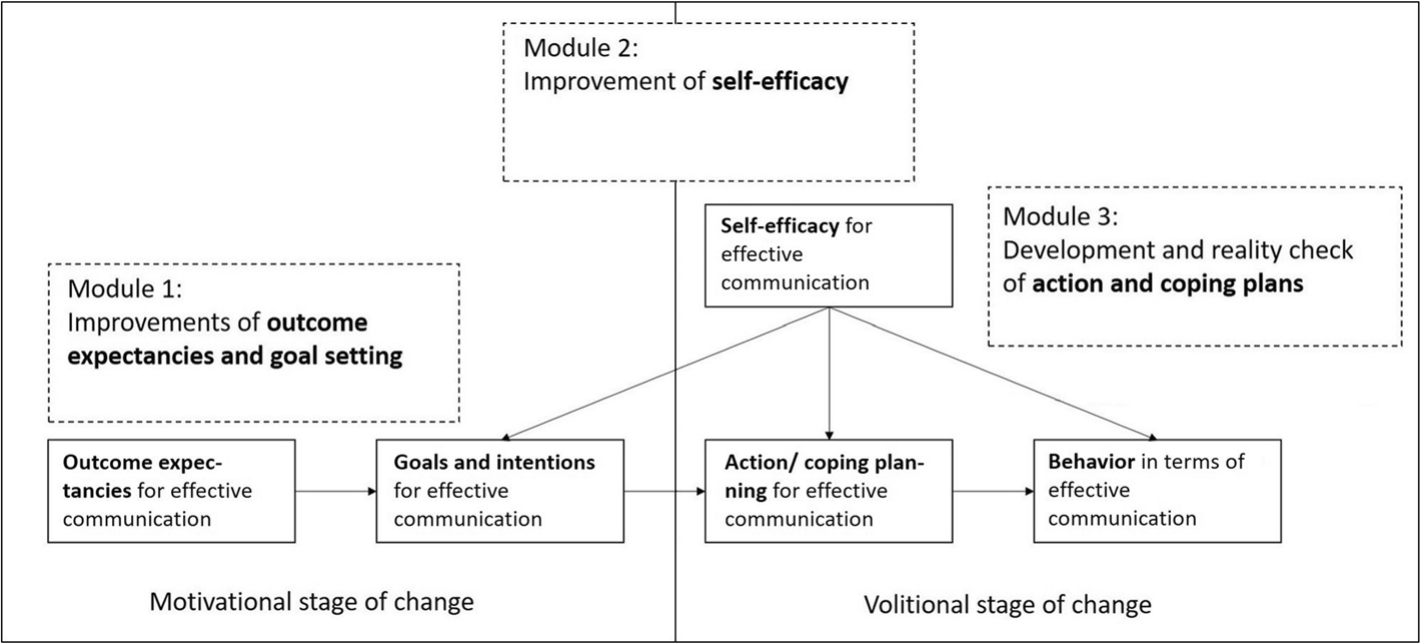
Components of the HAPA model and planned training modules for improving communication competences and adopting the behavior “effective communication”

While the models described above [33, 36] provide theoretical frameworks describing communication, they do not explain how effective communication behaviors can be implemented into daily practice. For instance, the inconsistency between knowledge about pain management and actual pain management described above begs the question why mothers are not treated more adequately if professionals know about the relationship between pain and patient satisfaction. The *Health Action Process Approach* (HAPA) [25] model describes how to translate the intention/motivation to adopt a new behavior into actually adopting the behavior, and specifically takes the role of motivation and awareness of a situation into account. The HAPA model has been found useful to describe, explain and successfully improve behaviors in a variety of settings, particularly in healthcare and preventive settings [38–42]. Modules for trainings and interventions can be developed and structured based on the HAPA model (Fig. 1).

The HAPA model suggests a distinction between the (a) *motivational phase* that leads to a behavioral goal intention/motivation, followed by the (b) *volitional phase* that leads to the actual health behavior [41]. Within the two phases, different patterns of social-cognitive predictors may emerge (see Fig. 1). In the initial motivation phase (a), a person develops an intention to act. In this phase, *risk perception* is seen as a distal antecedent (e.g., “I am at risk for poor communication”). Risk perception in itself is insufficient to enable a person to form an intention. Rather, it sets the stage for a contemplation process and further elaboration of thoughts about consequences and competences. Similarly, positive *outcome expectancies* (e.g., “If I communicate effectively, I will reduce my risk for adverse events and being unsatisfied”) are most relevant in the motivation phase when a person evaluates the pros and cons of certain behavioral outcomes. One needs to believe in their capability to perform a desired action, which is conceptualized as *perceived self-efficacy* (e.g., “I am capable to communicate effectively in spite of the high time pressure or difficult communication partners”). Perceived self-efficacy operates in concert with positive outcome expectancies, both of which substantially contribute to forming an intention. Both beliefs are needed for forming intentions to adopt difficult behaviors, such as effective communication. After forming an intention, the volitional phase (b) is entered.

When a person is inclined to adopt a particular health behavior, the ‘good intention’ has to be transformed into detailed (self-)instructions on how to perform the desired action by means of planning [43]. Once an action has been initiated, it has to be maintained. This is not achieved through a single act of will but involves ongoing use of self-regulatory skills and strategies. Thus, the volitional phase should be further broken down into more proximal factors, such as planning, self-efficacy, and social support. *Social support* is one factor reflecting the barriers and resources as part of the HAPA model: Support is a resource, and the lack of it can be a barrier to adopt or maintain health behaviors [42]. Instrumental, emotional, and informational social support can enable the adoption and continuation of behaviors. Social support can be addressed by means of directly integrating important network partners. For patients in gynecology and obstetrics, this network does not only include the professional at the clinic but also their social support providers such as the spouse or partner, key family members, friends, or the attending midwife who might not be employed with the hospital [9, 20]. Therefore, a *dyadic perspective* focusing on both the patient and their social support providers should be taken. For healthcare professionals, an intervention that uses a structured and applicable approach to enhance communication might help them to communicate effectively under a heavy workload. On the one hand, negative effects of impaired physician well-being on communication might be avoided; on the other hand, it is possible that communication becomes less challenging, which relieves stress. This has been shown in the context of nursing homes [44]. We therefore aim to include stress management in the HAPA model for healthcare professionals as a training-related outcome. Multiple studies explain the usefulness of the HAPA model to describe and promote behavior and behavior change (e.g., [45] including compliance [42], hand hygiene [46, 47] and vaccination [48]).

### Research questions and hypotheses

The interventions in this study will integrate the HAPA model and SACCIA-inspired communication competencies into an advanced training delivered face-to-face in groups of clinical staff, patients and their social support providers. In addition to this, a state-of-the art intervention will make use of digital options by translating the training material into an app [39, 40]. Previous research has shown that patient coaching can be an intervention to improve communication [49–51], and professionals can also significantly benefit [52, 53].

The *research questions of this project* are the following: what is the effect of a communication training delivered face-to-face and via a digital app on effective communication behaviors? To what extent can patient and healthcare provider satisfaction be improved and pAEs be reduced by improving effective communication behaviors? “Effective communication” is defined and operationalized in this project within the "SACCIA" framework [10, 17]. Beside these main outcomes, personal (e.g., perceived stress, coping) and training-related outcomes (e.g., goal intentions, action planning) will be assessed (see Table 1).

**Table 1:** Overview of the addressed concepts and hypotheses (numbers in brackets represent the hypotheses as described in text below)

	Phase 1	Phase 2	Phase 3
Communication competences (1., 4., 7.)	↑	↑	↑
Preventable adverse events (pAEs) (3., 6., 9.)	↓	↓	↓
Healthcare provider satisfaction (2., 8.)	↑	↑	↑
Patient satisfaction (5., 8.)	–	↑	↑
Training-related outcomes^1^ (outcome expectancies, goals and intention, action planning, coping planning, behavior, self-efficacy) (10.)	↑	↑	–
Personal outcomes (stress, coping, subjective safety culture) (10.)	↑	↑	↑
Organizational outcomes (adherence to safety culture) (11.)	↑	↑	↑

*Note:* ^1^As described in the HAPA model; ↓ = Hypotheses that criteria decreases due to the training; ↑ = Hypotheses that criteria increase; − = no evaluation planned

This will be scientifically evaluated using a three-phase study, in which phases are built sequentially on each other and described in detail in the method section: Phase 1, *implementation phase* (including needs assessment, retrospective cohort study of pAEs) *and pre-experimental study* with a group and pre−/post-test testing the effect of a training for professionals; Phase 2, *quasi-experimental efficacy study* with a randomized controlled trial study design (RCT) testing the effect of a training addressing patients and their social support providers; Phase 3, implementation of the app, aimed at staff, patients, and their social support providers, which will test the effect in *a case-control study*. Efficacy indicators are outlined in Table 1.

The *hypotheses* are the following: for Phase 1, it is expected that as a result of the training (1.) the communication competences of healthcare providers will measurably improve. As a result, (2.) healthcare providers will be more satisfied (3.) and the number and severity of pAEs should be significantly reduced for short and medium term (i.e. over the period of the intervention study and beyond). In Phase 2, it is expected that patients and their social support providers in the intervention group will develop significantly (4.) higher competences in communication with staff as well as (5.) higher satisfaction after the intervention compared to patients and their social support providers in the control group. It is also expected that (6.) the number and severity of pAEs in the intervention group will be significantly reduced compared to the number and severity of pAEs in the control group. For Phase 3, we hypothesize that the intervention group using the app compared to the intervention group without the app will show (7.) higher patient and healthcare provider communication competences and (8.) satisfaction as well as (9.) a significant reduction in the number and severity of pAEs. The effects of the training on communication competences may be (10.) mediated by training-related and personal outcomes and moderated by organizational factors (11.). All interventions in all phases will improve organizational outcomes if communication competences are improved successfully. Thus, the hypotheses will be tested in this project.

There are three aspects that make this project particularly unique. At first, previous studies have not combined the *underlying theories of communication and HAPA,* and tailored the intervention to the healthcare context. Secondly, the utilization of a *digital app* is expected to increase participation and training effectiveness. Thirdly, the project addresses healthcare providers, patients and their social support providers, regarding *all* people involved in developing effective communication and public health.

## Methods/design

**Fig. 2:**
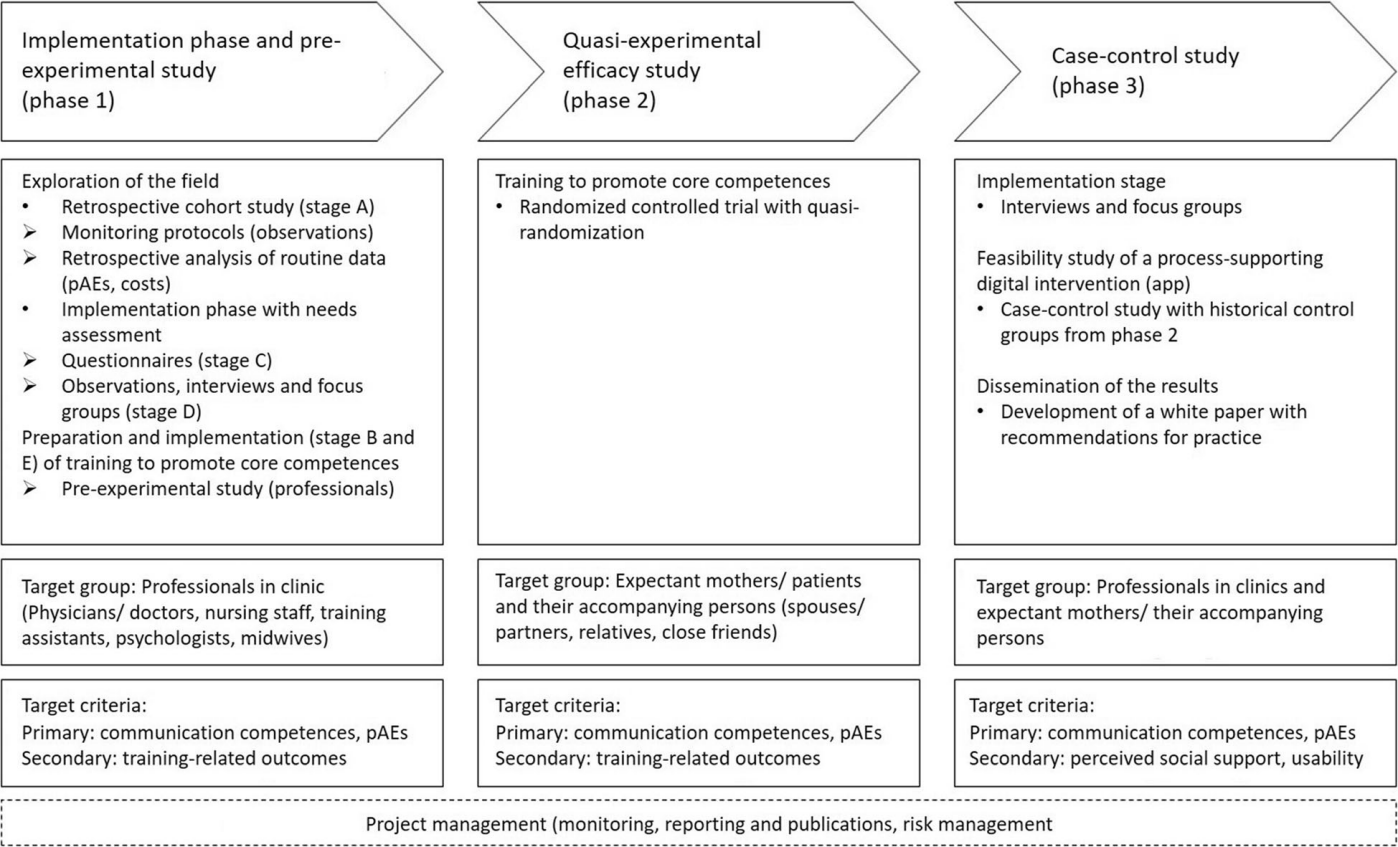
Study flow with project phases and stages, study participants and outcome (Study Flowcharts for all three Phases are in the Appendix). *Note.* The phases consist of different stages (see Table 3). Stages A to C (see Table 3) will be addressed in Phase 1. Stages D and E will be addressed in Phase 1 and 3

The project will apply a step-wise study design with three study phases in two level one perinatal maternity clinics: *Implementation phase and pre-experimental study* (Phase 1), a *quasi-experimental efficacy study* (Phase 2), and a *case control study* (Phase 3; Fig. 2).

For the study flow of enrollment and interventions for each phase, see the Appendix. All study participants are professionals, patients and social support providers in two participating gynecology and obstetrics clinics, therefore, they receive the regular treatment as patients in terms of healthcare or as staff with human resource (HR) management. Table 2 provides a summary of outcome variables across the study phases.

**Table 2:** Overview over the outcome variables and covariates considered in the different study phases

	Phase 1		Phase 2		Phase 3	
	T1	T2	T1	T2	T1	T2
Primary outcomes						
Communication competences^1^	X	X	X	X	X	X
Preventable adverse events (pAEs)^2^	X	X	X	X	X	X
Secondary outcomes						
Healthcare provider satisfaction^3^	X	X	X	X	X	X
Patient/treatment satisfaction^3^ (patients/social support providers)			X	X	X	X
Training-related outcomes^4^	X	X	X	X	X	X
Personal outcomes^5^	X	X	X	X	X	X
Organizational outcomes						
Adherence safety culture^6^	X	X	X	X	X	X
Subjective safety culture^7^			X	X	X	X
Covariates						
Socio-demographic variables including migration status^8^	X		X		X	

*Note:* Examples for the measurements are:

^1^ Self-developed questionnaire

^2^ Operationalized via trigger events (such as unavailable staff, equipment failure, readmissions, length of stay, communication error) as defined by [54, 55]

^3^ Nurses’ job satisfaction scale [56]

^4^ HAPA questionnaire including outcome expectancies, goals and intentions/motivation, action planning and coping planning, behavior, self-efficacy, perceived stress see [25, 42, 45–48]

^5^ Emotional exhaustion, depersonalization, perceived social support [4, 57]

^6^ The Hygiene Inventory - 23 items (HI-23) [58]

^7^ Measures equivalent to the ones used in [59, 60]

^8^ Age, gender, education, professional experience, depressive symptoms, anxiety and migration [61, 62]

### Phase 1: implementation phase and pre-experimental study

#### Overview of research questions and methods

For Phase 1, we conduct a needs assessment with regard to overall patient safety, based on the approach proposed by van Sluisveld et al., which aimed to improve the safety of patient transfers in intensive care units [63]. In the current project, this approach will be applied to gynecology and obstetrics clinics with their staff, patients (expecting mothers/women who are about to deliver or who gave birth recently), and social support providers (e.g., spouse/partner, relatives, close friends¸ Fig. 2). The approach differentiates into stages A to E (see Table 3), which will be conducted accordingly in the current study.

**Table 3:** Implementation phase: Research questions, methods, study participants and target criteria with regards to patient safety

Stage	Research questions	Methods	Target group/data source	Outcome measures
A	What is the status of communication competences?	Questionnaire (self-developed)	Healthcare providers	Communication competences as described by the SACCIA framework
	What is the prevalence of pAEs?	Routine data Mixed Methods study, Analysis of birth protocols (observations), staff questionnaire	Anonymous routine data of the hospital from the last year (2018), birth protocols and patient records, subjective prevalence	Quality indicators: pAEs such as unavailable staff, equipment failure, readmissions, length of stay, communication errors
B	What are effective interventions to improve safety and communication in everyday hospital life?	Scoping review	Pubmed, PsychInfo, Cochrane Database Web of Science Core Collection database^1^	Overview of effective interventions and effect sizes
C	What is the adherence for current patient safety measures (e.g., hand hygiene)?	Questionnaire, e.g., HI-23	All professionals at both intervention sites	Adherence to patient safety measures; relationships between adherence and quality indicators incl. patients’ satisfaction with their treatment and professionals work satisfaction
D	What are the resources and barriers for the implementation of an intervention in order to optimize communication in everyday hospital life?	Ethnographic observation; Individual semi-structured and focus group interviews	Physicians, nursing staff, training assistants, psychologists, midwives	Resources and barriers classified according to: (1.) intervention characteristics; (2.) societal context, (3.) implementation characteristics, (4.) institutional characteristics, (5.) social context, (6.) professional characteristics, and (7.) patient characteristics.
E	What is an appropriate strategy to implement effective interventions to optimize communication?	Intervention mapping with the method of Bartholomew and Kok (2011), triangulation of results from previous stages	Matching of data from interviews, focus groups and questionnaires with evidence from the literature	Implementation strategy tailored to the found resources and barriers to the implementation of effective interventions to increase patient safety

*Note:* pAEs = preventable adverse events; the content of this table is based on Table 2 in [63]

^1^The literature search for this paper used the following search term combinations:

Communication Training/Intervention AND Resource AND Implementation AND Health Experts

Communication Training/Intervention AND Barrier AND Implementation AND Professionals/Patients

Communication Training/Intervention AND Resource/Barrier AND Outcome Expectancies

We always maintained “Communication, Training, Intervention, Resource, Barrier” as search terms and will refine the larger searches with the following terms: Intention, plan, behavior, social-cognitive

Observations for the ethnographic analyses will be recorded using a standardized observation protocol [30]. The qualitative data will be analyzed in terms of inductive content analysis and will be used to develop the training. The details of this approach are described in Sluisveld et al. (2013) [63]. Focus groups and interviews will be conducted with partially standardized guidelines with at least one person from each occupational group in order to gain an impression of potential resources and barriers as comprehensive as possible (as found by previous research, e.g. [6]) for the implementation of the intervention [22]. *Immigration background* will be considered during data collection and testing as well as during app development (i.e. it will be ensured that patients and social support providers with an immigration background will also be included in the development as cultural background was found to influence communication behaviors [9, 61, 62, 64]).

This approach will lead to a comprehensive understanding of factors that facilitate and inhibit effective communication and pAE’s. Additionally, it will identify possibilities and potential benefits for the implementation of the intervention in the field, e.g., how communication competences can be integrated into clinical practice beyond the training. Possible options include daily communication logs, regular e-mails with tips on practical application etc.

The results will be used to develop the *training for professionals* (physicians, nursing staff, training assistants, psychologists, midwives), which will be implemented in Stage E of Phase 1 in this project (*pre-experimental study*; see Fig. 4 in the Appendix). The results will also be used to develop the *training for patients and their social support providers* in a *quasi-experimental efficacy study* in Phase 2 (see Fig. 5 in the Appendix). An equivalent procedure applying and testing a training program was successfully performed in a recent study [12]. However, only staff members were addressed and no communication competences or HAPA variables were targeted. In the current study, due to theoretical, ethical and methodological reasons, all individuals should get the benefit of the training. All professionals will be assigned to the intervention group and the evaluation will be done by a pre/post comparison.

#### Translation of the HAPA model into practice – development of the training

The design of the communication training will be informed by theory and previous empirically tested trainings. Exhibiting effective communication will be the desired behavioral outcome as specified in the HAPA model (Fig. 1). Performance modeling, performance desensitization (stemming from work on fears and anxiety disorders), performance exposure and self-instructed performance are all good methods for designing treatments that target enactive mastery experience [37, 38], or maintain the desired behavior in the HAPA model. An example would be to instruct persons to monitor and record how many times a day they have performed effective communication in face of time pressure and to track this on paper (Phase 1 and 2), or in the app (Phase 3). All of the above elements will be integrated into the trainings for professionals (in Phase 1, see Table 4) and for patients and their social support providers (in Phase 2, see Table 5).

**Table 4:** Contents and planned structure of the training for professionals (in Phase 1)

*1st part*	*2nd part*
Introduction and warming up; preview learning goals and reflection on expectations	Case studies and analyses with practical exercises and discussion
Training Part 1: Introduction in communication and patient safety	*Module 2*: Improvement of self-efficacy
Training Part 2: Previous experiences with communications skills/challenges	Training Part 3: Further work on communication competences
*Module 1*: Improvements of outcome expectancies and goal setting	*Module 3*: Development and reality check of action and coping plans
Transfer, reflections and feedback	Closing meeting with rounding up, further transfer exercise
Active break with networking and social support

**Table 5:** Contents and planned structure of the training for patients and their social support providers (in Phase 2)

*1st day*^*1*^	*2nd day*
Introduction and warming up; preview learning goals and reflection on expectations	Introduction and warming up
Training Part 1: Introduction in communication and patient safety	*Module 2*: Improvement of self-efficacy
Active break for networking and social support	Active morning break
Training Part 2: Previous experiences with communications skills/challenges	Training Part 3: Further work on communication competences
*Module 1*: Improvements of outcome expectancies and goal setting	Active break for networking and social support
Active break for networking and social support mobilization	
Case studies and -analyses with practical exercises and discussion	*Module 3*: Development + reality-check of action and coping plans
Transfer, reflections and feedback	Closing meeting with rounding up further transfer exercise

*Note*: ^1^Patients and their social support providers receive the training during two mornings. If participants are interested in an advanced training, another session will be provided

Training for professionals will take place during work hours and will last approximately 4 hours. Training for patients will last approximately 8 hours. The concrete development of the intervention content and procedure, in addition to the above description, is an important step towards employing *participatory intervention development within this first study phase*. The subsequent publications on the actual intervention contents and procedures will contribute to the current paper and allow for the replication of this study, including the interventions.

#### Procedure, sample, and data collection

*Training for professionals at the clinics* will integrate the communication competences and will be designed based on the HAPA model. A *short manual* will be provided to support the participants in implementing the lessons they learn into their working practice. Written documentation of the training will also be provided to avoid failure of implementation. The training will be offered to all professionals at both intervention sites. Its effectiveness will be tested in the pre-experimental study, which aims to investigate the association between training participation and the reduction/occurrence of pAEs, to observe the mechanisms that make the training successful with regard to supporting professionals in their work and understanding how patient training can be implemented in Phase 2.

The study (Stage E of Phase 1) has a one-group, pre−/post-design (see Fig. 4 in Appendix). It includes all professionals (e.g., doctors, nursing staff, midwives/obstetricians, training assistants, psychologists) at all locations and intends to recruit a total of *N* = 140 participants without any participant exclusion criteria. Professionals will be trained in interdisciplinary groups of 10 to 12 participants. Recruitment will take place via the hospitals involving line managers, works councils, quality management departments, and HR departments. They will be involved to ensure adequate participant enrollment to reach target sample size. Participants will be included in the analysis only if they provide informed consent, which will be collected by project managers working at the hospitals. Data will be analyzed according to intention to treat. If study participants withdraw their consent to be contacted for follow-up measurements at any point, their contact details will be removed from the database. If they indicate that they do not want their data included in the analyses, their data will also be removed. Otherwise, they will be considered as study dropout. All target criteria are shown in Table 2.

#### Statistical analyses

Training effects will be tested by evaluating differences between the measurement times using linear and general mixed models. Baseline values of the first measurement time point will be considered as covariates. Clinics are modeled as fixed effect. Key demographic variables such as age, gender and occupation will be used to calculate selectivity in dropout rates. Missing data per measurement point, but also over time, will be treated by advanced methods to handle missing data such as the full information maximum likelihood method (FIML; this will be the same in all other phases, too).

### Phase 2: quasi-experimental efficacy study

#### Aim/overview of research questions and methods

In Phase 2 (see Fig. 2 and Fig. 5 in Appendix), the effectiveness of the communication competences intervention for the target group of patients and their social support providers will be tested using the gold standard, i.e. a randomized control trial (RCT). This intervention will be based on all stages of Phase 1 of this project, and thus will be developed in a participatory approach with patients and professionals working in the clinics [63]. The intervention will enable patients and social support providers to gain knowledge about the prevalence of pAEs and communication problems as well as expectations of action results. Moreover, it will allow participants to practice communication competences and develop their self-efficacy. Furthermore, the intervention will utilize the provided theoretical input in order to integrate theory into everyday hospital life by means of action and coping plans. To implement the acquired competences as effectively as possible, the following three components will be addressed in the intervention [65]:

1. the motivation or intention to communicate effectively and confidently with each other,
2. the knowledge of what is crucial for effective communication,
3. the ability to communicate effectively.

The intervention is based on the HAPA model as a social-cognitive model for behavioral change [25] and communication competences (see description of the training above in Phase 1). Participants will be trained in groups by specialized instructors over the course of one and a half days in facilities of the participating clinics (see Table 5, and  Fig. 5 in Appendix).

#### Procedure

In each hospital, four *professionals will be taught in a train-the-trainer seminar* to train patients and their social support providers based on the short manual developed to teach communication competences. Recruitment will take place via the doctors, nurses and midwives in the hospitals (Fig. 5 in Appendix). The quality management departments will be involved to ensure adequate enrollment in order to reach the target sample size of N = 423 participants. Informed consent will be collected by on-site project managers. When an expectant mother is admitted to the clinic and agrees to participate in the study, she will be randomly assigned to a training group (IG) or a control group (CG). If she is accompanied by a partner, relative, or close friend, they will be assigned to the same group. All patients and social support providers randomly assigned to the intervention group will receive the same training content over the course of this study. In contrast, the control group will receive the hospital care-as-usual (see Fig. 5 in Appendix).

*Randomization* will be done per site and day (block randomization). The central project coordinator will generate the allocation sequence. Depending on this sequence, all patients admitted to the hospital on that day will be allocated to either the IG or to the CG. Patients and staff will not be informed beforehand on which days allocation to the IG or the CG takes place to prevent biases. Instead, the on-site project manager at each hospital will receive a sealed envelope each day containing this information, enabling them to inform other staff involved as well as enroll and assign participants to the CG or IG treatment. This process allows trial participants, general care providers, outcome assessors and data analysts to remain blinded throughout the allocation sequence. Trial participants and general care providers will be unblinded in case they ask for more information about the training. Revealing a participant’s allocated intervention during the trial can be performed after completion of the IG treatment to disclose the content of the intervention. A flyer will be provided containing this information, which will be also used after the study to disseminate findings and tools. Inclusion and exclusion criteria are outlined in Table 6.

**Table 6:** Summary of the inclusion and exclusion criteria

*Inclusion criteria*	*Exclusion criteria*
Expectant mother or *patient* in gynecology and obstetrics or *social support providers* (spouse/partner, relative, close friend) to be recipient of the training *Professional* in a gynecology and obstetrics hospital to be trainer of the patients and the social support providers	Not proficient in the German language and/or does not have the capability of writing Severe cognitive deficits (unable to read/write/answer questions) and impairments due to diagnosed brain injuries, neurological disorders, etc. Insufficient corrected eyesight (patients must be able to read on the cell phone) Participation in another research study or intervention trial conducted in the clinic
Aged 18 years and above	Younger than 18 years
Healthy volunteers	High risk, emergency case
Declaration of consent for participation in the study	Withdraw of consent for participation in the study at any point in time

According to a power analysis, a sample of *N* = 352 is necessary (alpha error = 0.05, power = 80%, to be analyzed IG _analysis_: *N* = 176; CG _analysis_: *N* = 176) in order to detect an effect size of *d* = 0.3. With an assumed drop-out rate of 20%, *N* = 423 participants (IG _recruited_: *N* = 212; CG _recruited_: *N* = 211) have to be recruited in the study. The pAEs will be collected on an individual basis (anonymized) and coded according to the communication competences (see Fig. 5 in Appendix).

#### Statistical analyses

The target criteria (see Tables 1 and 2) will be evaluated statistically and clinically, as in Phase 1, with linear and general mixed models and with *superiority trial tests* to investigate whether the intervention is better than the standard care. As expecting mothers and their social support providers participate in the intervention, dyadic data will be assessed and analyzed. Additional analyses will test whether subgroups of patients with psychological risk factors (e.g., depressive symptoms, anxiety) and low motivation benefit from the intervention to the same extent as study participants without such risk factors. Furthermore, adjusted analyses will be performed in terms of ANCOVAs considering factors related to social inequality, such as education, anxiety, and immigration status (see also Table 2).

### Phase 3: implementation of the app and case-control study

#### Functions of the app

**Fig. 3:**
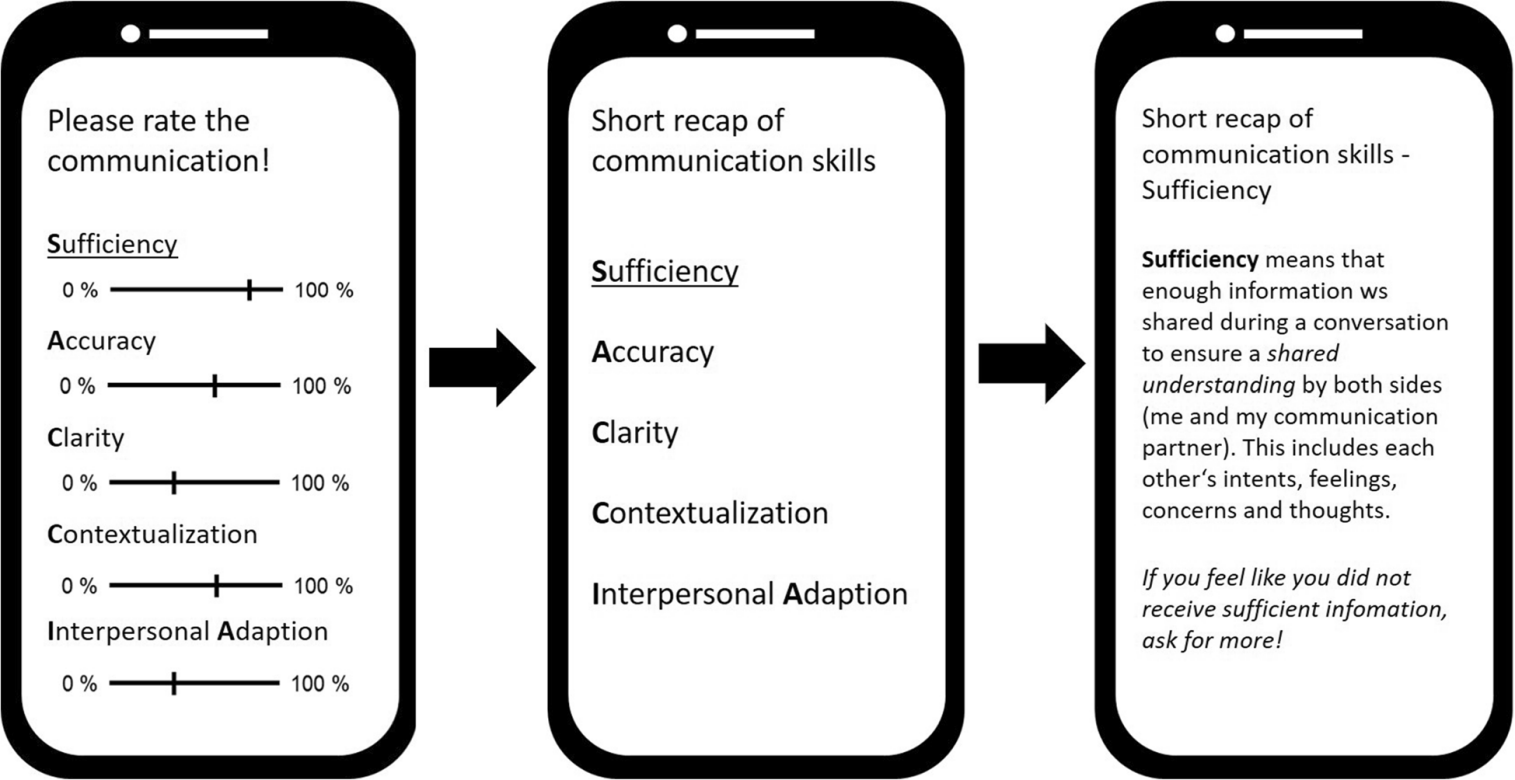
Example of the monitoring and guidance functions of the app

**Fig. 4:**
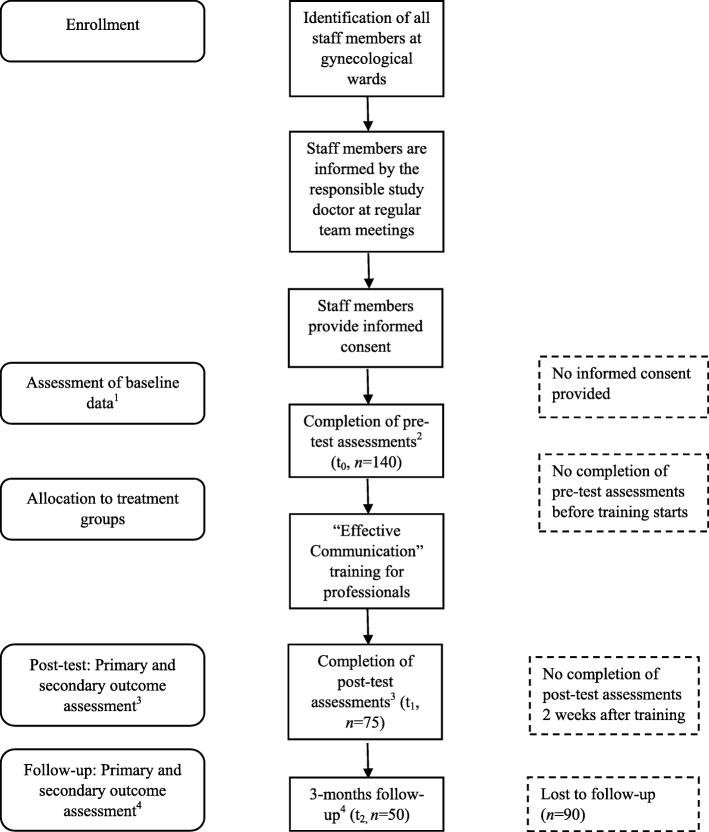
Trial design for Phase 1. Step 1: Participants (clinical staff members) are initially identified, informed and invited to take part in the training. Step 2: Participants give informed consent while baseline variables that can be measured independently are assessed. All participants in this phase receive the training so that there is no allocation to either treatment or control group but only to similar treatment groups. Step 3: Pre-tests^2^. Step 4: Participants complete the communication training. Step 5: Post-tests^3^. Step 6: Follow-up after 3 months. Note: ^1^ Baseline data includes baseline assessments of pAE prevalence and costs, adherence safety culture and job description. ^2^ Pre-test assessment includes demographic data, competences, training-related outcomes and work satisfaction. ^3^ Post-test assessments include competences and training-related outcomes. ^4^ At three months post-intervention, job stress and satisfaction, competences, behavior change as training-related outcomes and adherence safety culture will be assessed.

**Fig. 5:**
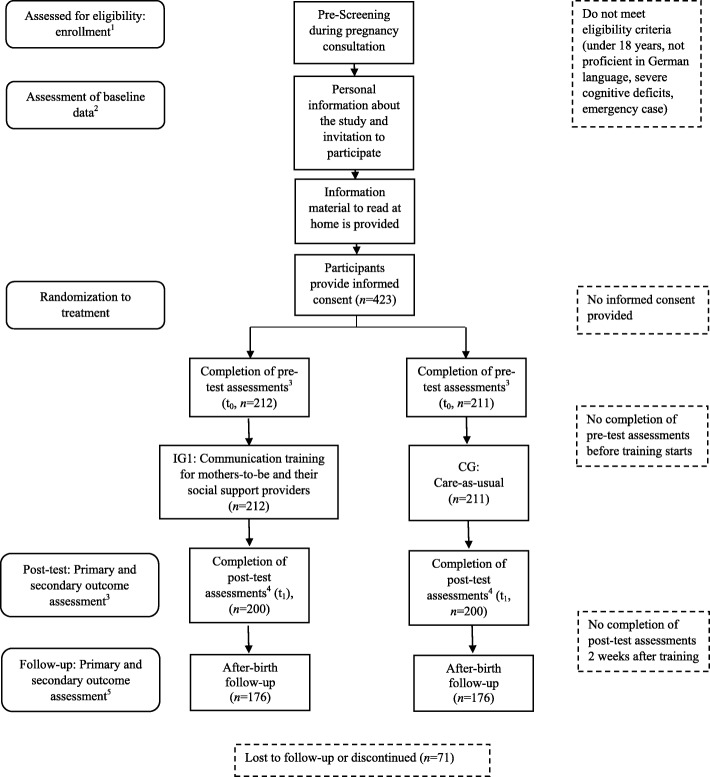
Trial design for Phase 2. Step 1: Participants (mothers-to-be/patients and their social support providers) are initially identified and screened for eligibility. Step 2: If eligible, participants are invited to take part in the study, give informed consent and are allocated to either the intervention group (IG, Communication training) or the control group (CG). Step 3: Pre-tests. Step 4: Participants in the intervention group complete the communication training. Step 5: Post-tests. Step 6: pAEs are assessed after the patient gave birth. *Note:* ^1^ For inclusion and exclusion criteria, see Table 6. ^2^ Baseline data includes baseline assessments of pAE prevalence and costs. ^3^ Pre-test assessment includes demographic data, perceived social support, competences, training-related outcomes, subjective safety culture and treatment satisfaction.^4^ Post-test assessments include perceived social support, competences and training-related outcomes. ^5^ At three months post-intervention, perceived social support, competences, behavior change as training-related outcome, subjective safety culture and treatment satisfaction will be assessed

A digital app will be developed to help users communicate well. The app will be developed in a participatory and theory-driven way from all findings and conclusions obtained in Phases 1 and 2 (Fig. 2) [66]. Stages D and E of Phase 1 will be repeated with professionals, expecting mothers/patients, and social support providers to update the evidence and answer questions that may have arisen during Phase 2. The aim of Phase 3 is to determine exactly how the app can support communication between professionals, patients, and their social support providers in their daily work/hospital experience and thereby lead to a reduction in pAEs. As app users will have completed the face-to-face training, they can use the app at their convenience to monitor their behavior and experiences (see Fig. 3) and deepen their skills and knowledge. To achieve this aim, there will be two functions of the app.

Firstly, the app will be designed to provide guidance on how to cope with specific (future) communication problems including communication initiated by a) the patient, b) the professional and c) between professionals. The following scenarios illustrate how the app may improve communication. Regarding a), if a woman prepares for a conversation with a professional in a labor ward, she may worry about how to express the wish for peridural anesthesia or more anesthesia if the pain escalates (cf. [21]). The app explains/provides suggestions to her (and her social support providers) how to communicate this wish in a clear and constructive way [43, 51] (Fig. 3). This may enable *shared decision making* in terms of understanding risks and disadvantages. Furthermore, she is supported to ask the right questions or maybe even audio record her questions and the answers from professionals, as it has been done in previous research [49]. Regarding b), a doctor may be unsure how to handle the expectant mother’s fear of childbirth and postpartum posttraumatic stress disorder (cf. [67]). Typical problems include sharing bad news and dealing with difficult emotions [24, 68, 69]. The app would help the doctor to communicate accurate information in a way that still addresses the fear that may come along with bad news. This may prevent complications due to insufficient information. Professionals will be asked to analyze and solve scenarios that involve both patients and their social support providers. The app will then explain the communication competences again and give concrete suggestions how to use them for solving the case. Depending on demands, the app will give suggestions such as “Speak slowly”, “Try not to use acronyms or abbreviations”, “Try to refrain from using scientific terms”, “Make use of visualizations”, “Be sensitive to verbal and non-verbal cues that may indicate lack of understanding”, “Stress the most important aspects that the patient must keep in mind”, and “Make use of the teach-back method” [19]. Regarding c), one team member of the delivery ward knows that a high-risk patient in labor needs prophylactic negative-pressure wound therapy (NPWT) after cesarean delivery, but they do not know how to communicate this to colleagues under time pressure [20]. The app helps to overcome time pressure by providing a message on the display for the colleagues which can be copied to a piece of paper, and thus support communication [70]. All of the above cases will be provided to colleagues, who will be asked to analyze and solve them. Communication competences will be explained with regard to communication between colleagues. Depending on demands, specific suggestions such as communication tools (daily goal sheet, bedside whiteboard, or door communication card), trust building, mindfulness, and reflective exercises will be given [71].

Secondly, the app will assist in monitoring typical or recent communication with focus on (1.) one’s own role, (2.) the role of the communication partner and (3.) resonance (a feeling of mutual understanding), thus supporting the development of general communication competences. These aspects will be evaluated with regard to the communication competences (see Tables 1 and 2).

Learning from the communication of all participants is ensured by collecting dyadic self-reported data and the partners reported data. Concretely, target-group specific tasks that train general communication competences seldom aid to overcoming specific obstacles, so reminders of resources and application/transfer options will be provided in the app, too.

#### Procedure, sample and data collection

Patients will be recruited via doctors and midwives in the hospitals; recruitment of staff and collection of informed consent of both patients and staff will be conducted by the on-site project managers. Quality management departments will be involved to ensure adequate participant enrollment to reach the target sample size. The target criteria of the patient sample will be evaluated statistically and clinically as in Phase 1 and 2 (see Table 2). Group differences will be tested with linear and general mixed models and with *superiority trial tests* that investigate whether the intervention is helping more than the standard care in Phase 2.

#### Statistical analyses

**Fig. 6:**
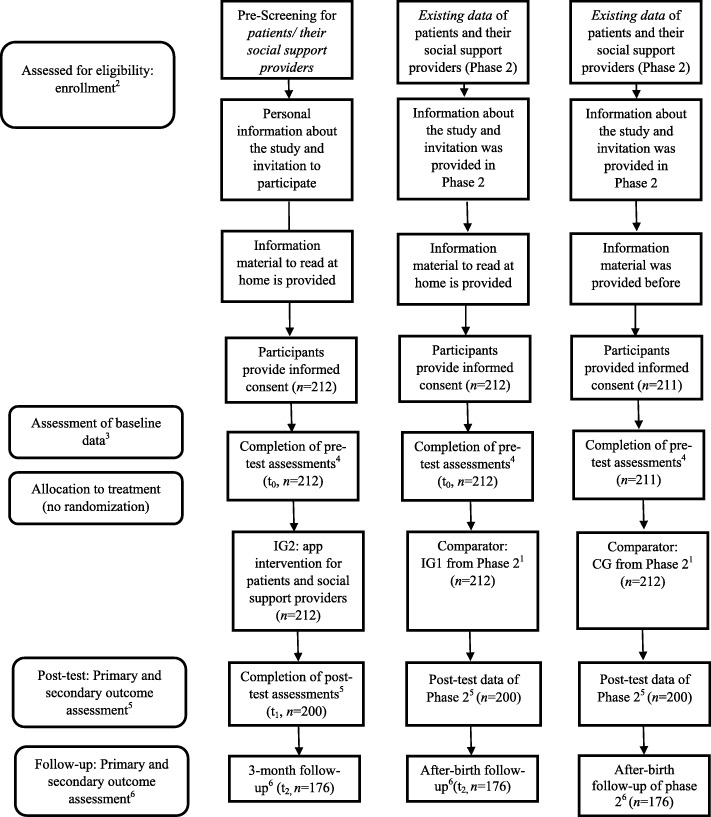
Trial design for Phase 3. Step 1: Participants (mothers-to-be/patients and their social support providers) are initially identified and screened for eligibility. All staff members are eligible for phase 3. Step 2: If eligible, participants give informed consent. Step 3: Pre-tests^4^. Step 4: Participants are provided with the Effective Communication App. Step 5: Post-tests^5^. Step 6: 3-months follow-up; person-specific pAEs are assessed after the patient gave birth. Possible reasons for participant drop-out have already been presented in Fig. 4/5. Note: ^1^ The comparator groups do not complete any tests again. Data acquisition in this group will be finished during Phase 2. ^2^ For inclusion/exclusion criteria, see Table 6. ^3^ Baseline data includes baseline assessments of pAE prevalence and costs. ^4^ Pre-test assessment includes demographic data, perceived social support, competences, training-related outcomes, subjective safety culture and treatment satisfaction. ^5^ Post-test assessments include perceived social support, competences and training-related outcomes. ^6^ At three months post-intervention, perceived social support, competences, behavior change as training-related outcome, subjective safety culture and treatment satisfaction will be assessed.

To test the effectiveness of the digital app, a case-control study will be performed (see Fig. 6 in Appendix). Cases are newly recruited individuals that fulfil the inclusion criteria outlined in Table 6. Comparators/controls are the participants in one of the two intervention arms of the previously conducted RCT study in Phase 2 (i.e. patients; historical control groups; No-Treatment CG _analysis_: *N* = 176; No-Treatment CG _recruited_: *N* = 212; Previously Treated/Active CG _analysis_: *N* = 176 (called IG1 in Fig. 6 in Appendix); Previously Treated/Active CG _recruited_: *N* = 212, see in section Phase 2). *N* = 176 participants need to be analyzed for the intervention group (IG2 _analysis_). Assuming a drop-out of 20%, *N* = 212 participants have to be recruited for the intervention group (IG2 _recruited_) in Phase 3 (see Fig. 6 in Appendix). The intervention will also be provided to the professionals in the clinic. Adoption and acceptance of the intervention will be evaluated in all groups by means of observational data and self-report. Target criteria are specified in Tables 1 and 2.

At the end of Phase 3, the results from all three phases will be disseminated using a white paper with recommendations for practice. Further plans to communicate trial results to participants, healthcare professionals, the public, and other relevant groups via publication, presentations and press releases will be developed using a participatory approach, with the restriction that anonymity is ensured at all times.

## Discussion

This project investigates interpersonal communication based on communication competences and the Health Action Process Approach (HAPA) to better understand where and how problems may occur and how to overcome these problems with interventions in everyday clinical life. Currently, there is no study that has done so in everyday clinical practice and has demonstrated the effectiveness of corresponding interventions based on the two theories as theoretical backdrop for designing the intervention.

The overall aim of the project is to support communication based on the communication competences model and the HAPA, and to overcome difficulties in everyday hospital life. The project is characterized by three innovative aspects. The first innovative aspect is the theoretical background and its application to maternity clinics. The *communication competences model* has been specifically designed to address communication in the healthcare context, whereas the *HAPA model* has been shown to bridge the gap between intention and behavior [25]. The combination of both HAPA and communication competences will aid in the design of an intervention specifically tailored to the healthcare context, which increases its potential effectiveness.

The second innovation of this project lies in its *digital focus, and thus, a focus on innovation policy*. The question is, to what extent can digital support of interpersonal communication actually support effective communication and which evidence-based recommendations can be given? Although there is some evidence, which we have reviewed in the beginning of this paper, this new project has a clear innovative potential to set the stage for future research in public health and prevention.

The third innovative aspect of this project lies in its systematic empirical investigation of *including professionals, patients and their social support providers* as active partners for patient safety. This adds to the value of this project by assessing dyadic data and is currently also regarded by the WHO as a central issue in patient safety [72].

Despite the need for developing healthcare professionals’ and patients’ communication skills, (ongoing) face-to-face training is rather time-consuming and may not be achievable given the overall need for increasing hospital efficiency [73]. Digital communication training offers a solution to this dilemma as it reduces costs and organization resources. Moreover, users can participate in the training according to their own schedules, thus potentially increasing their motivation and the training’s effectiveness, but only if the digital trainings are sufficiently implemented and monitored [74].

Two studies included in a previous review evaluated the effectiveness of training interventions to improve communication [9]. The training package targeting communication skills for doctors was not associated with higher satisfaction with work scores recorded by women, although they reported high satisfaction with training workshops [75]. While Crofts et al. reported that improvements in all their test variables in the three clinical scenarios were statistically significant after the training, this was based on a pre- and post-intervention analysis with no comparison groups [76]. Perceptions of safety and communication significantly improved after training with patient-actors, compared to training using mannequins for postpartum hemorrhage scenarios, but it is unclear why no statistically significant improvements were found. Furthermore, the authors revealed no benefits of additional teamwork training on patient-actor perceptions of care related to safety, communication, and respect [76]. Due to the innovative aspects outlined above, our own evaluation is expected to outperform these previous effects in order to support communication. This study will also provide valuable information on the effectiveness, user-acceptability, and feasibility of the intervention. It addresses the need to investigate new approaches to improve communication, which can relieve health systems from the growing demands caused by challenges with communication around the world and in all areas of medicine and public health.

## Data Availability

The full protocol including data security protocol (in German)  and publication guidelines will be available from the corresponding author on reasonable request. Data sharing is not applicable to this article as no datasets were generated or analyzed so far.

## Abbreviations

BCT: Behavior change techniques
CG: Control group
HAPA: Health Action Process Approach
IG: Intervention group
N/n: Number of study participants
NPWT: Negative-pressure wound therapy
pAEs: Preventable adverse events
RCT: Randomized controlled trial
SACCIA: “Sufficiency”, “Accuracy”, “Clarity”, “Contextualization”, and “Interpersonal Adaptation”
SBAR: “Situation”, “Background”, “Assessment”, and “Recommendation”
WHO: World Health Organization
